# Vitamin D status in longstanding type 1 diabetes and controls. Association with upper extremity impairments

**DOI:** 10.48101/ujms.v128.9888

**Published:** 2023-11-22

**Authors:** Hans J. Arnqvist, Per Leanderson, Anna Spångeus

**Affiliations:** a Department of Endocrinology and Department of Clinical and Experimental Medicine, Linköping University, Linköping, Sweden; b Department of Occupational and Environmental Medicine and Department of Medical and Health Sciences, Linköping University, Linköping, Sweden; c Department of Acute Internal Medicine and Geriatrics, Linköping University Hospital, Linköping, Sweden; d Department of Medical and Health Sciences, Linköping University, Linköping, Sweden

**Keywords:** Type 1 diabetes, vitamin-D, complications, upper extremity impairments

## Abstract

**Background:**

Patients with type 1 diabetes have a high prevalence of upper extremity impairments (UEIs), such as frozen shoulder, carpal tunnel syndrome, and trigger finger. The UEIs are strongly associated with activity limitations and impaired quality of life. The etiology of the UEI is not clear. Vitamin D deficiency has been considered to play a role in the pathogenesis of type 1 diabetes and in the development of macro- and microvascular complications in diabetes.

**Aim:**

To characterize vitamin D status in a large population of patients with type 1 diabetes, if vitamin D deficiency is associated with metabolic factors and possible association with UEI.

**Material and methods:**

Patients who diagnosed before 35 years of age, whose diabetes duration >20 years, and who are not older than 65 years were invited to participate in this cross-sectional case-control, multicenter study. Controls matched for age and sex were obtained from the national population registry. Fasting blood samples were collected and stored at −80°C until analyzed regarding 25-hydroxy-vitamin D (25(OH)D3) by a liquid chromatographic-mass spectrometric method (LC-MS/MS).

**Results:**

Vitamin D levels varied with season as expected in the northern hemisphere. The association between 25(OH)D3 and clinical variables was analyzed in a univariate general linear model, which indicated no difference in 25(OH)D3 in men with and without diabetes but higher values in women with diabetes. About 30% of both patients and controls had vitamin D deficiency (≤50 nmol/L). Analyzed by binary logistic regression UEIs was not associated with 25(OH)D3 levels. In both patients and controls, 25(OH)D3 was correlated to apolipoprotein A1 (*r* = 0.153; 0.220, *P* < 0.001).

**Conclusion:**

In patients with type 1 diabetes and a duration of 20 years or more, vitamin D level is not lower than in nondiabetic controls and is not associated with UEIs.

## Introduction

Vitamin D has been associated with type 1 diabetes for several reasons (1). Type 1 diabetes is an autoimmune disease, and its development is influenced by environmental factors (2). Vitamin D is a known immunomodulator and is considered to promote immune tolerance and to have immunosuppressive properties (1, 3). Low levels of vitamin D have been reported at diagnosis of type 1 diabetes in children and adults (4, 5). Several but not all studies have shown lower D-vitamin levels in type 1 diabetes than in controls without diabetes (6, 7). A potential role of vitamin D deficiency in the development of macro- and microvascular complications in diabetes has been suggested (8). Vitamin D supplementation appears to have a beneficial effect by reducing serum total cholesterol, Low density lipoprotein (LDL) cholesterol, and triglyceride levels but not High density lipoprotein (HDL) cholesterol levels (9).

Patients with type 1 diabetes have an increased prevalence of upper extremity impairments (UEIs), such as frozen shoulder, carpal tunnel syndrome, and trigger finger (10). In a population-based study of patients with type 1 diabetes and long duration, we recently showed that the prevalence of UEIs is 2–3-fold higher than that in matched controls without diabetes (11). These musculo-skeletal complications have strong impact on daily activities and quality of life (11, 12).

This study had three aims: 1) to investigate if vitamin D deficiency is more prevalent in patients with type 1 diabetes and long duration than in matched nondiabetic controls; 2) if vitamin D deficiency is associated with metabolic factors; and 3) if vitamin D deficiency is associated with UEIs.

## Material and methods

### Design and inclusion

This cross-sectional, case-control, multicenter study was performed in cooperation with all nine hospitals in the Southeast region in Sweden between 2010 and 2013. Using the hospitals local diabetes register, all patients in the region with type 1 diabetes diagnosed before 35 years of age, not older than 67 years, with a diabetes duration ≥20 years were invited to participate. Invitation was done by a postal letter with a questionnaire enclosed. Controls matched for sex and age ±5 years were obtained from the Swedish population register. When a patient accepted to participate, the corresponding controls were invited. Controls were excluded if reporting diabetes or if laboratory tests showed an elevated fasting plasma glucose level ≥7 mmol/L.

### Questionnaire

A self-administered questionnaire containing four parts was sent to the patients. Controls received the same questionnaire except for diabetes specific questions. In the present study, we only included data from the first part of the questionnaire, which was study specific and has been described in a previous publication (11). In brief, this part of the questionnaire contained issues on background characteristics, such as weight, length, sex, diabetes duration, occupational habits, presence of diabetes complications, and UEIs. The presence of UEIs (self-reported) was categorized in five groups: 1) shoulder pain and stiffness, 2) tingling and numbness in fingers and/or wakening in the night due to pain and/or numbness in hand or fingers, 3) hand stiffness, 4) finger lock phenomenon when bending a finger, and 5) flexed finger defined by inability to extend one or several fingers. There were also questions regarding previous surgery of carpal tunnel syndrome and trigger finger. The other parts of the questionnaire (data not used in the present study) included the health assessment questionnaire (HAQ); the disabilities of the arm, shoulder, and hand (DASH); and the Short Form 36 Health Survey (SF-36).

### Participant characteristics

Altogether 773 patients with type 1 diabetes and 708 matched nondiabetic controls were included by answering the questionnaire (11). The participants were asked to provide blood samples, and 603 patients with type 1 diabetes and 531 control subjects delivered blood samples. For this study analyzing 25(OH)D3, 586 blood samples from patients and 518 samples from controls were available. Compared to the patients, the control group included more women (64% vs. 56%, *P* < 0.05) and was older (55 ± 9 vs. 51 ± 10, *P* < 0.001). The patients had a mean diabetes duration of 36 ± 10 years. As reported earlier (11), all five impairments were significantly more prevalent in patients compared to controls, that is, shoulder pain and stiffness 40% versus 17%, hand paresthesia 48% versus 28%, hand stiffness 36% versus 15%, finger locking 32% versus 12%, and flexed finger 30% versus 6%.

### Drop out analysis

Drop out analysis has been reported in a previous publication (11). Of all the type 1 diabetes patients invited (*n* = 1,727), 773 patients accepted participation and were included in this study. In total, 708 controls accepted participation and were included (*n* = 1,995 invited).

### Laboratory measures

Patients and controls were asked to take a blood sample at their local hospital, and results have been published previously except for Apolipoprotein A1, Apolipoprotein B, and alanine amino transferase (ALAT) (11, 13). Blood sampling was done by venepuncture after an overnight fast. All blood samples were analyzed as routine measurements at the Department of Clinical Chemistry, Linköping University Hospital. The laboratory is accredited by SWEDAC (Swedish Board for Accreditation and Conformity Assessment). Plasma glucose was analyzed using an Advia 1200 instrument (Siemens Healthcare Diagnostics). HbA1c was analyzed with the TOSOH G7 automated hemoglobin analyzer (Tosoh Bioscience, Tokyo, Japan). Controls were also asked to take a blood sample in the same manner as the patients except for HbA1c, which was exclusively analyzed in patients.

For analysis of 25-hydroxyvitamin D2 and D3, the plasma samples were worked up according to the method described by Turpeinen et al. (14) before derivatization with 4-phenyl-1,2,4-triazoline-3,5-dione and finally analyzed by high-performance liquid chromatography-electrospray tandem mass spectrometry. Quality of the assay was assured by participation in the Vitamin D External Quality Assessment Scheme (15).

### Ethics

An informed signed consent was obtained from all the participants. The Research Ethics Committee of the Faculty of Health Sciences, Linköping University, approved of this study (M245-09:2010-03-17).

### Statistics

Mean and standard deviation were reported for continuous variables. Student’s t-test was performed when comparing two groups and Analysis of variance (ANOVA) using Bonferroni as a post hoc if there were three or more groups. Chi-square test was used for categorical variables. Pearson’s correlation analysis was used to study possible associations with clinical variables. Normality of clinical variables was tested by histogram and normality plots. Only GFR, ALAT, and IGFBP-1 had a somewhat skewed distribution, but correlation coefficients and *P*-values were about the same with Pearson and Spearman’s correlations.

To account for the differences in age and sex between patients and controls, univariate general linear regression was used with 25(OH)D3 as the dependent variable, patient and controls and sex as categorical variables, and age, body mass index (BMI), GFR, ApoA1, ApoB/ApoA1, ALAT, glucose, and IGF-1 Z-score as independent variables. The association of UEIs with 25(OH)D3 levels was analyzed by binary logistic regression.

All statistical tests were performed at the 5% significance level. Statistics were calculated using SPSS 23.0 for Windows software (IBM Statistics, New York, USA).

## Results

**Figure 1:**
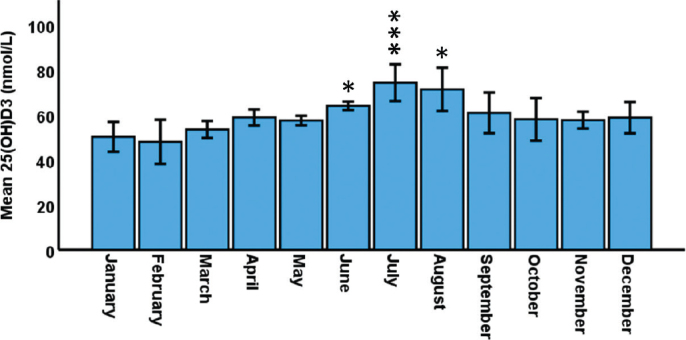
Mean 25(OH)D3 distribution by month for the whole cohort (patients + controls). *P < 0.05 and ***P < 0.001 compared to January and February.

The monthly averages of 25(OH)D3 levels in all participants (with or without diabetes) are shown in Figure 1. There was a clear seasonal variation with lowest levels observed in January-February and the highest in July and August. Compared to January, the 25(OH)D3 levels were significantly higher during June to August.

We also measured 25(OH)D2, but this variant was typically not possible to detect, or if so, the levels were less than 1 nmol/L, and the inclusion of 25(OH)D2 had therefore no effect on the results presented in Figure 1.

**Figure 2:**
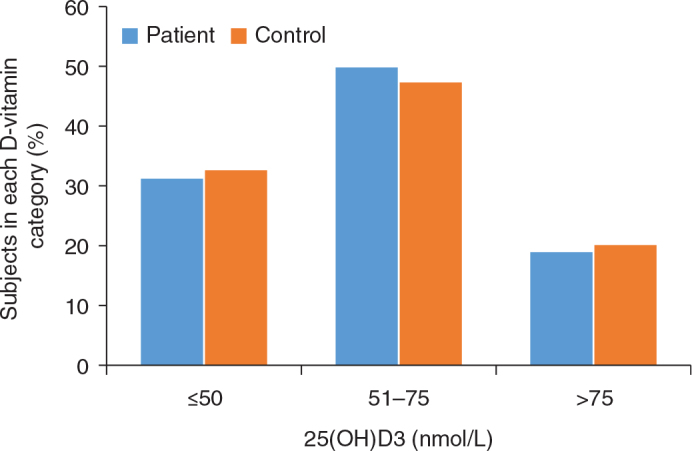
Distribution of patients with type 1 diabetes and controls without diabetes in 25(OH)D3 categories ≤50, 51–75 och >75 nmol/L. There were no significant differences.

Neither the mean values, for the whole year, independent of month taken, differ between patients (60 ± 19) and controls (59 ± 19), *P* = 0.640, the mean difference being 0.53, 95% CI −1.7; 2.8, nor the prevalence of vitamin D insufficiency (≤50 nmol/L) differs, 31.2% versus 32.6%, *P* = 0.651 (Figure 2). There were no significant differences between patients and controls when 25(OH)D3 was categorized into ≤50, 51–75, and >75 nmol/L.

Since there were significant differences between patients with type 1 diabetes and controls regarding age and sex, we used a univariate general linear model to study possible interactions between group factors and clincal parameters (Table 1). There was a significant difference for 25(OH)D3 between patient and control groups (*P* = 0.011) and a significant interaction between patients/controls and sex (*P* = 0.021). When analyzing men and women separately, a significant difference for 25(OH)D3 between patient and control group was only found for women (*P* = 0.029), indicating lower 25(OH)D3 values in women in the control group.

**Table 1:** Univariate general linear model with 25(OH)D3 as dependent variable. Parameter estimates.

Parameter	All subjects		Women		Men	
	B Coeff. (95% CI)	*P*	B Coeff. (95% CI)	*P*	B Coeff. (95% CI)	*P*
Age (years)	−0.157 (−0.300; −0.014)	0.032	−0.259 (−0.444; −0.075)	0.006	0.009 (−0.225; 0.244)	0.938
BMI (Kg/m^2^)	−0.339 (−0.631; −0.046)	0.023	−0.511 (−0.876; −0.146)	0.006	−0.009 (−0.505; 0.487)	0.971
GFR (ml/min)	−0.154 (−0.283; −0.076)	<0.001	−0.179 (−0.284; −0.075)	<0.001	−0.124 (−0.247; −0.001)	0.048
ApoA1 (g/L)	11.714 (6.463; 6.965)	<0.001	13.360 (6.558; −0.075)	<0.001	10.311 (1.769; 18.854)	0.018
ApoB/ApoA1	0.846 (−7.202; 8.894)	0.837	1.704 (−9.220; 12.628)	0.759	1.887 (−10.366; 14.140)	0.762
ALAT (µkat/L)	−4.927 (−9.238; −0.615)	0.025	−3.940 (−10.463; 2.583)	0.236	−5.695 (−11.547; 0.157)	0.056
Glucose (mmol/L)	−0.696 (−1.059; −0.333)	<0.001	−0.699 (−1.178; −0.220)	0.004	−0.711 (−1.274; −0.147)	0.014
IGF1 Z-score	0.037 (−0.790; 0.865)	0.929	0.153 (−0.868; 1.175)	0.768	−0.270 (−1.707; 1.167)	0.712
Controls	−4.936 (−8.729; −1.142)	0.011	−4.767 (−9.037; −0.496)	0.029	0.022 (−5.322; 5.365)	0.994
Patients	0^a^	.	0^b^		0^b^	.
Men	−0.792 (−4.120; 2.535)	0.641	.			.
Women	0^a^	.				
Men * Controls	5.584 (0.855; 10.312)	0.021	.	.		.
Men * Patients	0^a^	.	.	.		.
Women * Controls	0^a^	.				
Women * Patients	0^a^	.	.			

ALAT: Alanine Amino Transferase; Apo: Apolipoprotein; GFR: Glomerular filtration rate; CI: confidence interval; BMI: body mass index.

a This parameter is set to zero because it is redundant.

b This parameter is set to zero because it is redundant.

### Association of 25(OH)D3 with metabolic factors

BMI, GFR, lnCRP, IGFBP-1, and IGF-1 Z-score in patients and controls have been published previously (11, 13). Data on ALAT activity, apolipoprotein A1, apolipoprotein B, and apolipoprotein B/apolipoprotein A1 quotient are given in Table 2. ALAT activity did not differ significantly between patients with type 1 diabetes and controls without diabetes. Compared to nondiabetic controls, apolipoprotein A1 was significantly higher, and apolipoprotein B and the ratio Apolipoprotein B/Apolipoprotein A1 were significantly lower in patients with type 1 diabetes (Table 2).

**Table 2:** Apolipoprotein concentration and alanine amino transferase activity in patients and controls.

Variable	Type 1 diabetes	Control	*P*
ALAT (µkat/L)	0.45 (0.30)	0.47 (0.22)	NS
ApoA1 (g/L)	1.54 (0.30)	1.50 (0.26)	0.007
ApoB (g/L)	0.90 (0.19)	1.10 (0.22)	<0.001
Ratio ApoB/ApoA1	0.60 (0.16)	0.76 (0.20)	<0.001

ALAT: Alanine Amino Transferase; Apo: Apolipoprotein.

The association of 25(OH)D3 values with selected metabolic factors was tested by bivariate Pearson correlations (Table 3). In patients with type 1 diabetes, lower 25(OH)D3 was significantly correlated to lower apolipoprotein-A1 and IGF-1 Z-score, and higher ALAT activity, GFR, and plasma glucose. In controls, lower 25(OH)D3 was significantly correlated to lower apolipoprotein-A1, and higher BMI, GFR, lnCRP, IGFBP-1, and apolipoprotein-B/ apolipoprotein-A1 quotient.

**Table 3:** Pearson correlations between 25(OH)D3 and clinical parameters in patients with type 1 diabetes and controls.

Variable	Type 1 diabetes		Control	
	*r*	*P*	*r*	*P*
Age (year)	0.047	0.257	0.006	0.883
BMI (Kg/m^2^)	−0.055	0.190	−0.195	**<0.001**
GFR (ml/min/1.73m^2^)	−0.078	0.068	−0.219	**<0.001**
lnCRP (mg/L)	−0.071	0.100	−0.112	**0.013**
IGFBP-1 (µg/L)	−0.064	0.123	0.152	**<0.001**
ApoA1 (g/L)	0.153	**<0.001**	0.220	**<0.001**
ApoB (g/L)	0.059	0.172	−0.015	0.733
ApoB/ApoA1	−0.071	0.099	−0.139	**0.002**
ALAT (µkat/L)	−0.117	**0.006**	−0.073	0.104
Glucose (mmol/L)	−0.137	**0.001**	−0.61	0.178
IGF-1 Z-score	0.094	**0.027**	−0.019	0.681
HbA1c	−0.078	0.070	-	-
Duration	0.073	0.080	-	-

BMI: Body mass index; GFR: Glomerular filtration rate; lnCRP: logarithm C-reactive protein; IGFBP: insulin-like growth factor-binding protein; ALAT: Alanine Amino Transferase; Apo: Apolipoprotein; IGF: insulin-like growth factors.

Significant P-values are shown in bold italics.

### Upper extremity impairments and 25(OH)D3 levels in type 1 diabetes

To find out if there was any association between UEIs and 25(OH)D3 levels, we explored the association using binary logistic regression (Table 4). Shoulder impairment, hand paresthesia, hand stiffness, finger locking, or flexed finger were included as binary variables, and 25(OH)D3, patient/control, age, sex, BMI, GFR, ApoA1, ApoA1/ApoB, ALAT, and glucose as independent variables. The only tendency to an effect was found for hand paresthesia with a *P-value of 0.108 while P-values for the other UEIs were far from significant*.

**Table 4:** Association of upper extremity impairments with 25(OH)D3 in patients with type 1 diabetes and controls analyzed by binary logistic regression.

Parameter	Shoulder impairment		Hand stiffness		Hand paresthesia		Finger locking		Flexed finger	
	OR (95% CI)	*P*	OR (95% CI)	*P*	OR (95% CI)	*P*	OR (95% CI)	*P*	OR (95% CI)	*P*
25(OH)D3 (nmol/L)	1.002 (0.994–1.010)	0.675	0.999 (0.991–1.007)	0.756	0.994 (0.986–1.001)	0.108	1.003 (0.994–1.012)	0.497	0.996 (0.986–1.005)	0.358
Pat./Contr.	4.003 (2.637–6.076)	<0.001	3.706 (2.420–5.676)	<0.001	3.048 (2.087–4.451)	<0.001	2.803 (1.789–4.391)	<0.001	6.007 (3.567–10.116)	<0.001
Age (years)	1.017 (1.002–1.033)	0.023	1.048 (1.028–1.069)	<0.001	1.015 (0.998–1.032)	0.089	1.040 (1.019–1.062)	<0.001	1.059 (1.035–1.084)	<0.001
Female/Male	2.161 (1.555–3.002)	<0.001	2.168 (1.545–3.043)	<0.001	2.041 (1.503–2.772)	<0.001	1.486 (1.049–2.104)	0.026	1.231 (0.848–1.786)	0.275
BMI (Kg/m^2^)	1.040 (1.003–1.078)	<0.001	1.001 (0.965–1.039)	0.942	1.073 (/1.037–1.111)	<0.001	1.010 (0.971–1.050)	0.620	0.961 (0.920–1.003)	0.071
GFR (ml/min)	1.010 (1.000–1.020)	0.048	1.016 (1.005–1.026)	0.003	1.002 (0.993–1.012)	0.611	0.999 (0.989–1.010)	0.906	1.006 (0.995–1.017)	0.305
ApoA1 (g/L)	0.837 (0.434–1.616)	0.596	1.73 (0.551–2.088)	0.836	0.865 (0.466–1.605)	0.646	0.933 (0.467–1.865)	0.844	0.855 (0.405–1.805)	0.682
ApoA1/ApoB	1.996 (0.716–5.564)	0.186	1.142 (0.390–3.339)	0.809	0.976 (0.374–2.552)	0.961	0.605 (0.190–1.930)	0.396	0.739 (0.206–2.647)	0.642
ALAT (µkat/L)	!.525 (0.903–2.578)	0.115	1.157 (0.684–1.956)	0.586	1.552 (0.913–2.640)	0.105	1.300 (0.756–2.235)	0.342	1.162 (0.649–2.083)	0.613
Glucose (mmol/L)	1.024 (0.982–1.069	0.264	1.032 (0.989–1.077)	0.147	0.993 (0.953–1.034)	0.727	1.057 (1.012–1.104)	0.013	1.046 (1.000–1.093)	0.052

BMI: Body mass index; GFR: Glomerular filtration rate; ALAT: Alanine Amino Transferase; Apo: Apolipoprotein; OR: odds ratio; CI: confidence interval.

## Discussion

### Seasonal variation of 25-OH vitamin D

The concentrations of vitamin D do normally vary during the year due to different hours of sun exposure (16). In the present study, the circulating 25(OH)D3-values varied with the lowest values reached in winter (January-February) and the highest in the summer (July-August), which is in well agreement with the expected seasonal variation in the northern hemisphere.

### 25(OH)D3 level in type 1 diabetes and controls without diabetes

Our control group was matched according to sex and age ±5 years and were recruited by an invitation letter with a questionnaire included. However, the response rate varied with age and sex, which is why we ended up with significant differences in age and sex. A univariate general linear model, used to adjust for these differences, indicated that there was no difference in men regarding 25(OH)D3 levels, while women with type 1 diabetes had somewhat higher values than controls. In a meta analysis, Shen et al. (7) found no differenc in vitamin D levels between patients with type 1 diabetes older than 14 years and controls, which is in agreement with our results. In patients with type 1 diabetes residing in a solar-rich environement, reduced vitamin D levels were not associated with type 1 diabetes (17). A 25(OH)D3 level below 50 nmol/L is considered as D-vitamin deficiency based on the observation that lower values are associated with increased levels of parathyroid hormone (18). 25(OH)D3 levels below 50 nmol/L were found in about 30% of patients and controls with no significant difference.

In conclusion, our data suggest that in patients with type 1 diabetes and long duration D-vitamin levels are not lower than in nondiabetic controls, and there is no difference in the prevalence of D-vitamin deficiency.

### Association of 25(OH)D3 levels with clinical parameters

Apolipoprotein A1 was significantly higher in patients with type 1 diabetes – an observation in line with previous reports (19), and the Apolipoprotein B/Apolipoprotein A1 quotient was lower. High apolipoprotein A1 in type 1 diabetes may be due to exogenous insulin (20). It should be mentioned that according to clinical guidelines, a large number of the patients with diabetes were probably treated with statins, which may raise apolipoprotein A1 and lower apolipoprotein B (21). These observations are in favor of a reduced risk of atherosclerosis, which is somewhat surprising since patients with type 1 diabetes have an increased risk of atherosclerotic events (22). In both patients and controls, there was a positive association between 25(OH)D3 levels and Apolipoprotein A1 (*P* < 0.001). Vitamin D3 has been reported to be positively related to apolipoprotein A1 in both men and women (23). In type 2 diabetes, regular consumption of vitamin D in a randomized study was found to ameliorate apolipoprotein A1 (24). Hypovitaminosis D has previously also been associated with low apolipoprotein A1 (25).

### Upper extremity impairments and 25(OH)D3 levels in type 1 diabetes

Muscolo-skeletal complications are common in diabetes and can hamper daily life activities as well as quality of life. The patophysiology of these complications is not clear and probably multifactorial. Poor glycemic control is reported as a contributing factor in most but not all studies (10, 11, 26). We hypothesized that vitamin D deficiency could be a contributing factor to UEIs. In binary logistic regression, we found no significant association between D-vitamin levels and UEIs, which is why our study does not support a role for D-vitamin in the pathophysiology of UEIs.

The strength of our study is that we analyzed a large well characterized cohort of patients with type 1 diabetes and a control group. 25(OH)D was analyzed simultaneously in patients and controls by a high-quality mass spectrometry-method with external quality assessment. Limitations of our study are that the time for blood sampling was not matched between patients and controls, and the controls were somewhat older, and there were slightly more females.

## Conclusion

Our data from this large population-based study suggest that in patients with type 1 diabetes and over 20 years durations, D-vitamin levels are not lower than in nondiabetic controls and are not associated with UEIs. In both patients and controls, Apolipoptrotein A1 is positively associated with vitamin-D levels.

## Funding

This work was supported by the Medical Research Council of Southeast Sweden (FORSS) under grant number FORS-660161 and Stiftelseförvaltningen Region Östergötland, Sweden.

## Disclosure statement

The authors have nothing to declare.

## Notes on contributors

HA and AS designed the study, HA, AS, and PL gathered data and performed statistical analysis, PL completed the biochemical analysis, and HA wrote the first draft of the manuscript. All authors have read and agreed to the published version.
